# Notes and Comments

**Published:** 1921-10

**Authors:** 


### HOSPITAL and HEALTH REVIEW

### NOTES AND COMMENTS.

THIS is the first number of a new Review. It carries on the tradition of the *Hospital*, and reviews, in addition, the vast subject of the health of the people and the prevention of disease. In mid-Victorian days it was fashionable "to enjoy bad health"; but in these present times few, indeed, of us can afford that luxury. Good health is the greatest asset that a man or woman can possess, and ill-health, on the contrary, often means bodily and financial ruin. The appreciation of these facts to-day accounts for the growing interest that is taken in matters of preventive medicine and of public health not only by the medical profession, but also among the laity.

IN THE HOSPITAL AND HEALTH REVIEW we shall attempt to give our readers a critical and reasoned survey of recent advances in public-health affairs, retaining at the same time articles on matters of hospital interest. It is not every public-health officer, or general practitioner, or health visitor, or matron, or nurse who has access to recent public-health literature, or has the time and inclination to read much of it. In this Journal we intend, however, to review important events and literature in such a manner that THE HOSPITAL AND HEALTH REVIEW will rightly be considered to be a sufficient summary and criticism of current public-health affairs.

WE shall be pleased to receive articles, annotations, and other contributions from the readers of this paper, and to hear any news from hospitals or public-health departments about matters affecting their well-being. Our contributors and correspondents should send us their names and addresses, not necessarily for publication, and should enclose stamped and addressed envelopes if they desire manuscripts to be returned. Our correspondence columns, according to the space available, are open to everyone, although obviously we cannot be held responsible for the opinions expressed by those who write us letters for publication. Finally, we shall be pleased to consider criticisms of, and suggestions for, this Review.

HOSPITALS and Health Departments are alike affected by the straitened financial circumstances that prevail at the present time. Prices are high and money is scarce, and as a result some hospitals have reduced their expenditure by closing wards, and the health authorities likewise are forced to spend less money. To what extent it is true economy not to spend money on health is debatable, but we believe, for example, that it might have been cheaper and easier to prevent the cholera and typhus in Russia and Poland rather than to allow millions of people to suffer and hundreds of thousands to die. We hope that we shall never see economy grown into parsimony over matters that affect vitally our physical well-being. A universal pestilence throughout England would bring us to financial and national disaster.

THE scarcity of money is likely to affect adversely the co-operation which was slowly growing between the health services and the voluntary hospitals in England. For many years the Public Health and School Medical Services have been making more and more use of the general hospitals in their districts. Many local authorities reserve beds for maternity cases, for ailing and sick babies, and for school children in the local hospitals, and pay suitably for this accommodation. Cases of venereal disease, of tuberculosis, and of other conditions of which the local health authority has the care are often provided for in this manner. We believe that where this co-operation has existed the results have been

**NOTES AND COMMENTS**—(*cont.*).

altogether admirable, and we should regret if the wave of "economy" that is passing over the country were to lessen any of this valuable work. It may not, perhaps, be the moment to increase these activities; but certainly we should be unwise if we attempted to go back to the bad but cheaper conditions that prevailed ten or fifteen years ago.

---

We see, without very much surprise, that the Treasury grant to local authorities, in respect of milk that is given to babies and nursing mothers, will be substantially reduced. This reduction of Government expenditure on milk is partly the result of the "economy" programme; and in part is due to the fact that some local authorities distribute milk in an unbusinesslike and extravagant manner. There are a few districts where large expenditure on milk is incurred; and no steps are taken to ascertain that the recipients of the free milk are really in need of it. Ratepayers in these spendthrift areas can wash in the municipal milk if they desire: no inquiries are made into the financial circumstances of the applicants; if they ask for free milk, well! that is enough, let them have it. So these wasteful authorities use up the Government subsidy, and other people have to suffer. If only all local authorities had been reasonably careful, there would be enough money to go round; but as it is the amount of grant has to be curtailed. We hear, often enough, about *panem et circenses* in the days of decadent Rome. To-day certainly we do not give bread, but we give milk, which is a better food; we have heard it argued that we have no free shows for the people, but indeed that is not the case. The ratepayers can attend, free of charge, the meetings of their local authorities, and in some of these spendthrift areas we believe these meetings are better than any circus.

---

The hospitals and the maternity centres of the health departments have been busy, as usual, this season with the babies who are stricken with "summer diarrhœa." Unfortunately there have been many cases and many deaths. Of the babies who do not die some are left permanently damaged, and grow later into the stunted and poorly-nourished children whom we see later in the elementary schools. Both the incidence and the fatality of summer diarrhœa have decreased during the past few years, first, because of the anti-fly campaign waged by the local medical officers of health, and, secondly, on account of the valuable work of the maternity and child-welfare centres and their associated health visitors; but, although there has been an improvement, yet we have still much to do before we relegate this ineffective enteritis to the limbo of forgotten diseases.

---

In the summer weather the temperature of the ground rises until it becomes favourable (given suitable conditions of moisture also) for the rapid breeding of flies. As long as the soil temperature keeps high, the flies stay out of doors; when the earth temperature falls, in come the flies, and bring the diarrhœa with them by infecting milk and food. That, shortly, is a description of the mechanics of summer diarrhœa infection. Of the nature of the infecting organism we are ignorant; we even doubt if there is any one specific germ. So many different micro-organisms have been ascribed as the cause of the disease that we must either believe in them all as the cause, or else discredit all of them. The intestinal tract of a little child is relatively free from anærobic sporing bacilli, and it is, at any rate, credible that a dose of such bacilli, found always in horse manure where the fly breeds, may be sufficient to cause an acute enteritis—in other words, that summer diarrhœa is really a soil disease.

---

The abnormally dry summer resulted, in many places, in a shortage of water; but there was no acute water famine in any of our larger towns. The country villages and isolated houses dependent upon rain- or well-water were more severely affected by the prolonged drought. The Ministry of Health have recently issued a short and timely circular, in which the chlorination of doubtful water supplies is recommended. The chlorination of drinking-water was practised extensively among our armies during the war, and resulted in the saving of innumerable lives. Several towns in England have been chlorinating their drinking-water for many years past, and in America the process is almost universal. The chlorination of drinking-water is cheap and is a certain method of sterilisation; but, in addition, it has this great advantage—that it is rapid, and it is applicable at short notice to water sources great or small—to the well of a cottage or to the water supply of a great town.

---

The Man in the Street, who not so long ago was in the trenches, objected greatly to the water in the battalion water-cart. Often it was unutterably vile, especially when the battalion medical officer did not himself drink of it habitually. It was difficult to enjoy a drink of that over-chlorinated water, and certainly sometimes it set the teeth on edge. Present-day methods for the treatment of public supplies have none of those disadvantages, and there are several admirable and fool-proof chlorination plants on the market which make none of those mistakes of the enthusiasts in Flanders.

---

It is not uncommon for outbreaks of infectious disease to occur in hospitals, especially in children's hospitals, and for "cross-infections" to develop among the patients admitted with some other infective disease to an isolation hospital. Such infections and cross-infections, coming as they do to complicate a case which may already be severe, lead sometimes to terrible tragedies. Those who have ever seen an outbreak of measles among babies who are recovering from summer diarrhœa, or a scarlet-fever infection in a diphtheria ward, will know how tragic can be the results that follow on these mixed infections.

**NOTES AND COMMENTS—(*cont.*).**

In order to guard against the introduction of infective diseases into an orthopædic ward of a general hospital in Philadelphia, the following stringent precautions are taken. They might well be copied in our own children's hospitals. All children, on admission to hospital, are examined for any evidence of throat infection, rash on any part of the body, and, in the case of girls, a vaginal discharge. The parents are questioned with reference to any recent contagious disease in the home or school or among the child's playmates. Cultures are made from the nose and throat, and specimens taken from all girl children to be examined for vaginitis. If these latter specimens are pronounced negative by the laboratory, the child is admitted to a probation room for five days. If the child has never been vaccinated, that is done during the first three days. Every child is immunised with diphtheria antitoxin during the same period. By the fourth day all the reports from the laboratory are returned. On the fifth day, after a careful inspection of the child's body, the child is transferred to the ward. Vaginal specimens are sent to the laboratory each week during the child's stay in the hospital. Every bed-pan is surgically clean every time it is used. This plan has been entirely successful, but necessarily occupies much of the time of the medical and nursing staff. It is time, however, that is very well spent, and these precautions should reduce to a minimum the chance of importing infectious disease into a hospital.

But it should be recognised that cases of infectious disease sometimes seem to occur sporadically, without any known source of infection being ascertained; and also that now and then an infectious disease seems to become endemic in a hospital ward—cases of infection occur at irregular intervals, and they cannot be definitely attributed to any previous case. The periodic disinfection of children's toys and of other fomites in a hospital is a potent factor in diminishing the incidence of these untraceable outbreaks of infection. The disinfection of the ward itself—once, perhaps, in three months—by means of formalin, seems also to check cross-infection.

The care of hospital floors is a matter which is often neglected. Of course the floors in hospitals always look very pretty. Sometimes they are so highly polished as to be dangerous; but there are few floors that will bear close inspection. Children love to sit and to play on the floors; they dig between the interstices of the boards or blocks, and prize the pieces of dust that they have succeeded in removing with the ward-maid's fallen hairpin. It is common for children in hospital to scratch their knees and to damage their fingers and hands with splinters. The greatest care in dealing with the individual on admission to hospital may be nullified if the floor of the ward and the ward itself is merely a store-house for dust, dirt, and infection. Dustless sweeping, an antiseptic floor-polish, and a floor surface without irregularities and crevices will prevent disease in many patients.

A short article appeared, not so long ago, in one of the weekly medical papers, in which the present-day position of the "family doctor" was called into question. Is there a family doctor, asks the writer of the annotation, any more than there is a family picture palace? Such a question is no doubt justified regarding our larger towns and dense centres of population; but in the smaller towns and in the country the family physician still holds his own. His father before him and his son after him doctor many generations of the same families, for the countryman is essentially conservative. The farmer may practise the rotation of crops, but he would not countenance a rota of practitioners. We think there is much to be said in favour of perpetuating the family doctor, for the patient grows no better but rather the worse in visiting a multitude of physicians. A peripatetic patient, who consults a dozen different doctors in a year, has no faith in any one of them, and that element of confidence which aids the cure is therefore lacking. And not one of the dozen practitioners is given a real chance, for none can obtain sufficient knowledge of his transitory patient—and this perpetual flux is not to the credit of the one or to the health of the other.

The public realises, from experience and the Sunday papers, that all doctors are not of equal merit, and that a brass plate is not necessarily a sign of competence in the art of healing. The hurried scurry from one doctor to another is evidence of the search for an ideal. When we are ill or those near to us are sick we do not demand a mediocre physician; we try to find the best that we can afford. It is generally admitted by doctors and by the laity that the medical education of the student is not all it should be, and on this subject we shall have more to say in a later issue.

The better training of the medical student and the institution of quinquennial post-graduate courses for all qualified medical men would do much to improve our present medical services and to allay adverse criticisms of the profession, especially if at the same time it became understood by the people that the modern physician takes a pride in preventing disease among his patients. If a practitioner of medicine has a thousand persons on his panel, and if these persons are quite well throughout the year, then that doctor makes a comfortable little sum of money without having to work for it. If, however, each of these thousand patients is ill once during the year, even with a trivial sickness, probably the doctor will have to give at least two thousand consultations. Not one of these thousand patients really wants to be ill, and certainly no doctor desires to add to his work; therefore, it is to the interest both of the patient and of the doctor that there should be no sickness. How great an amount of disability there is we read in the report of the Chief Medical Officer of the Ministry of Health for 1920. Sir George Newman writes: At least 14,066,000 weeks' work are lost on an average every year through sickness, or a period of upwards of 270,000 years. That is to say, in

**NOTES AND COMMENTS—(*cont.*).**

England and Wales there is lost to the nation every year, among the insured population only and excluding the loss due to sickness for which sickness or disablement benefit is not payable, the equivalent of the work of 270,000 persons. Moreover, it must be remembered that it is not only the working equivalent of 270,000 persons that the nation loses each year, but also the labour and expense involved in their care during the fourteen million weeks of their incapacitation."

---

Sir Arthur Newsholme, in an article on "Public Health Education," which we publish in the present number, says: "It is not sufficiently realised that at least one-half of our present mortality is avoidable; *i.e.*, could be postponed to old age when death becomes a normal event of life. With this unnecessary mortality goes an incredibly large amount of avoidable sickness."

---

A movement for the endowment of a number of beds has started at Nottingham, and is being pursued in an interesting way. Messrs. Players set the example to private businesses, but the impulse has not stopped there. The general staff of the Nottingham Post Office has started a scheme to raise the necessary £1,000 in twelve months, and this projected gift will be in addition to the weekly contributions which are made for the general maintenance of the hospital. It is a typical example of seeking to develop existing sources of income on a wider and better organised system, and as such may be commended to other hospitals which fancy that the familiar ways of raising revenue are exhausted.

---

The decision of the board of management of King Edward VII. Hospital, Cardiff, to appoint a medical superintendent is of great interest, and perhaps a significant sign of the times. The appointment was recommended by the Emergency and Reference Committee, and supported by Sir William Diamond as economical as well as useful. The new medical superintendent will have a salary of £650, and replace the resident house surgeon, who receives about £350. Our readers will recall that Mr. Leonard Rea, the present Secretary and superintendent, has long been troubled by his multifarious duties, and it seems clear that the appointment of a medical superintendent will be welcomed not least by himself. This point is worth remark for the benefit of institutions generally, since in hospitals south of the Tweed there has been a tendency to suppose that a medical and a lay superintendent were naturally incompatible officials. In our own advocacy of the medical superintendent we have had to guard against this misconception, and welcome the instance which Cardiff provides in support of our view. Of course, it must be remembered that the appointment of a medical superintendent at Cardiff coincides with the opening of the Medical School, and the matter is not perhaps so easy to determine when a non-teaching hospital is in question. Nevertheless, the change is noteworthy, and arrangements adduced in its support did not lay stress upon the Medical School. Rather they insisted upon the claims of the general superintendent to be left free to cope with his proper duties, and the advantages to the "hospital" side of the work when placed under a medical chief. An important and welcome departure has been made, the example of which should not be lost upon other institutions. Its success, however, would be more assured if the salary offered were such as to attract the most capable men.

---

There must be something extremely arduous in the task of being a "medical correspondent" to a lay paper. The public wants something rousing to read in the train on the way to the office, and something tasty to swallow on the journey home again in the evening. The medical correspondent has to provide literature to fill in the gaps between the news items of the paper—the battle, murder, and sudden death in which the readers really delight. It must be, mentally, disastrous and trying work; and internal evidence is not wanting in these pseudo-medical articles that the writers are dissatisfied with themselves and with their efforts. They even become bad-tempered in the intervals between the dishing up of hybrid science and the re-hashing, for the public consumption, of medical discoveries of long ago.

---

Many of our hospitals and Public Health Departments produce leaflets for the information and enlightenment of their people. Some of these leaflets are bad, many are moderate, and a few are good, in that they achieve the object for which they were designed. One of the worst leaflets that we ever saw emanated from a borough that prided itself on its low death-rate and excellent sanitary administration. This leaflet consisted of four foolscap pages, closely printed, with extracts from Public Health Acts, warning people of penalties which they might incur under various Acts and by-laws. The cost of printing this unreadable nonsense must have been enormous; and the householder, to whom it was distributed, read, no doubt, none of it. We are not enamoured of American propaganda methods; but a leaflet, both here and in America, must have some "snap" or "ginger" about it if it is to be read, and therefore to be successful.

---

Readers of this Review who will refer to page 29 will see that we are offering three guineas to the person who writes for us the best leaflet on "The Care of the Teeth." We consider that a well-designed leaflet on this subject might prove to be of use to schools and to education authorities. If we receive any really good leaflets in consequence of this competition we shall be prepared to publish them for circulation among the elementary, secondary, and private schools. We shall print the best leaflet in the next number of this Review. But those who try their luck in designing leaflets for us should remember that brevity is the soul of wit, and that children are very terrible critics!

